# Field experiment reveals that female Bechstein’s bats (*Myotis bechsteinii*) select bat boxes based on the space available for roosting

**DOI:** 10.1007/s00442-025-05700-9

**Published:** 2025-04-02

**Authors:** Christina Willemsens, Gerald Kerth, Jesús R. Hernández-Montero

**Affiliations:** Zoological Institute and Museum, Applied Zoology and Nature Conservation, Greifswald University, Loitzer Straße 26, 17489 Greifswald, Germany

**Keywords:** Day roosts, Bat conservation, Exploration, RFID-monitoring, Roost selection

## Abstract

Roosts are a crucial resource for bats, which choose them based on many factors, including the surrounding habitat, microclimate, and space available for communal roosting. The latter is important because many bat species benefit from social thermoregulation by forming colonies in their roosts. However, it remains unclear whether bats can base their roost choice directly on the space available for roosting when other characteristics, such as roost microclimate do not vary. We present results from a field experiment in which RFID-tagged Bechstein’s bats (*Myotis bechsteinii*) in a maternity colony were given paired bat boxes with identical external dimensions but differing internal roosting space. This allowed us to control for other factors that might influence roost choice and to record the bats’ nightly visits to the boxes prior to their occupation as day roosts. To assess whether roost temperature influenced roost choice in our setup, we measured the internal temperature of the boxes. Female Bechstein’s bats showed a clear preference for boxes with more roosting space, as evidenced by a higher frequency of nightly visits and more frequent use as day roosts. As there was no significant difference in temperature between boxes with different internal volumes, roost temperature cannot explain the bats’ preference for spacious roosts in our experiment. Our data provide evidence that bats can directly assess the volume of their roosts. Our results are consistent with the argument that small volume roosts may limit roosting group size potentially reducing social thermoregulation benefits.

**Supplementary Information:**

The online version contains supplementary material available at 10.1007/s00442-025-05700-9.

## Introduction

Roosts provide bats with protection from weather and predators, and serve as a place for mating, rearing young, and social interactions (Kunz 1982; Kunz and Fenton 2005). Therefore, the ongoing loss of roosting sites caused by human land use is one of the greatest threats to bats (Mickleburgh et al. 2002; Frick et al. 2020). Intense forest management, for example, reduces the number of old and dead trees with cavities that can serve as roosting sites, thereby negatively affecting bat populations (Mickleburgh et al. 2002). In addition to a sufficient number of roosts, bats also need roosts with certain characteristics. For example, maternity colonies often prefer warmer roosts because they are energetically favorable during gestation, lactation, and pup growth (Kerth et al. 2001; Sedgeley 2001; Bergeson et al. 2021).

To mitigate the loss of naturally occurring roosts in managed forests, artificial roosts (bat boxes) have been used as a conservation tool (Crawford and O’Keefe 2024). This practice has been used in Germany since the 1950s (Issel and Issel 1955). It has been shown that bat boxes fulfil the basic requirements of a roost for bats living in tree cavities, such as a suitable microclimate, protection from the weather and predators, and space for groups to form (Ruegger 2016). Bat boxes also provide a valuable tool for research (Rueegger 2016) as they allow to study population dynamics, behavior, and physiology, among other aspects (Kerth 2022). Furthermore, bat boxes are a popular tool for environmental education that increases public awareness of bats (Agnelli et al. 2011; Lear 2017).

Several factors influence the likelihood that bats roost in bat boxes, such as the surrounding habitat and box placement (López-Baucells et al. 2017), ambient temperature, box microclimate (Kerth et al. 2001), and their design (Rueegger et al. 2019; Pschonny et al. 2022). Box design plays a major role because it can be adapted to species-specific preferences (Baranauskas 2009) and may influence box microclimate (Tillman et al. 2021; Bakken et al. 2022). While wood is the most commonly used material for the construction of bat boxes in Asia, Australia, and North America, boxes made of wood-concrete are most frequently used in Europe (Mering and Chambers 2014; Rueegger 2016). Wood-concrete is a product containing 75% wood with temperature-regulating materials such as clay (Schwegler, Germany). Differences in box shape range from voluminous round boxes to flat square boxes (Mering and Chambers 2014; Rueegger 2016).

Previous studies have addressed species-specific preferences by deploying different box types. For example, brown long-eared bats (*Plecotus auritus*) preferred boxes with large internal volumes (Dodds and Bilston 2013). Indiana bats (*Myotis sodalis*) also preferred the largest available design of multi-chambered boxes (Hoeh et al. 2018). In contrast, boxes with narrow crevices are preferred by barbastelle bats (*Barbastella barbastellus*; Rachwald et al. 2018) and Brandt’s bats (*M. brandtii*; Pschonny et al. 2022). Other species, such as the common pipistrelle (*Pipistrellus pipistrellus*), are more flexible and use multiple box types as day roosts (Pschonny et al. 2022). For some species, roosting preferences can vary seasonally (Kunz 1982; Jankowska-Jarek et al. 2023). Finally, individual traits of bats such as age, sex, and reproductive condition can also influence the selection of roost types (Rueegger 2016). For example, female Bechstein’s bats (*Myotis bechsteinii*) selected roosts with different temperatures depending on their reproductive condition (Kerth et al. 2001). In general, however, artificial roosts show higher occupancy rates if they provide conditions similar to those found in the natural occurring roosts of the target species (Mering and Chambers 2014).

The material from which the boxes are made and their color can influence roost selection, as both can affect the temperature inside the roost (Kerth et al. 2001; Brouwer and Henrard 2020; Jankowska-Jarek et al. 2023). This demonstrates that design elements affecting the box’s microclimate can play an important role in roost selection due to their effect on the bats’ energy budgets and physiological processes (Tillman et al. 2021; Mundinger et al. 2023). Finally, the internal volume of roosts influences their occupation by bats (Dodds and Bilston 2013; Rueegger 2016; Pschonny et al. 2022). Dodds and Bilston (2013) showed that boxes with larger volumes are preferred and used by larger groups of Natterer’s bats (*Myotis nattereri*). This may be because larger volumes allow for the formation of larger roosting groups, allowing group members to benefit more from social thermoregulation (Kunz 1982; Willis et al. 2006; Willis and Brigham 2007). Social thermoregulation refers to the behavioral strategy of huddling, which reduces individual energy expenditure by 6 to 53%, depending on the number of individuals and the species involved (Gilbert et al. 2010). Although to our knowledge no study has examined the relationship between social thermoregulation and roost volume, previous studies have shown energetic benefits arise from communal roosting groups of big brown bats (*Eptesicus fuscus*, Willis and Brigham 2007), little brown bats (*Myotis lucifugus*, Webber and Willis 2018), Daubenton’s bats (*Myotis daubentonii*, Dietz and Kalko 2006), Bechstein’s bats (*Myotis bechsteinii*, Pretzlaff et al. 2010) and barbastelle bats (*Barbastella barbastellus*, Russo et al. 2017). Given the potential energetic benefits of communal roosting, roost volume has been proposed as an important roost selection criterion for colonial, forest-dwelling bats (Sedgeley and O’Donnell 2004, Willis et al. 2006). Finally, the volume of a roost can also directly influence its microclimate, with larger volumes offering more stable temperatures and a thermal gradient as in the case of taller boxes such as rocket boxes (Tillman et al. 2021).

The influence of roost volume on roost occupation has been indirectly addressed by comparing the rates at which different box types are used as day roosts (Hoeh et al. 2018; Rueegger et al. 2019). For example, Dodds and Bilston (2013) compared five different bat box types, differing in their volume. However, in these studies, the selection process leading to roost occupation was not investigated because the nightly visits of bats could not be monitored. Moreover, the influence of other box design elements other than volume was not controlled for because box types with different shapes and designs were used. Brittingham and Williams (2000) investigated how two bat box designs with the same internal volume but different thermal characteristics affected box use by displaced big brown (*Eptesicus fucus*) and little brown (*Myotis lucifugus*) bat colonies. Although both box designs were occupied, bats presumably benefited from boxes that provided a wide temperature gradient, helping them to reduce physiological expenditure. The same study found other factors such as the amount of solar radiation received and the prevalence of ectoparasites, also played a role in bat roosting preferences.

Here, we used a field experiment to investigate the influence of roost internal volume on roost choice in female Bechstein’s bats, controlling for confounding factors such as box shape, material, color, and the surrounding habitat. Bechstein’s bats live in maternity colonies that comprise 10–80 philopatric females, which communally roost in tree cavities and bat boxes (Kerth et al. 2001; Kerth and Van Schaik 2012). Colonies show fission–fusion dynamics, often splitting into subgroups that use separate day roosts (Kerth and König 1999). Because female Bechstein’s bats change their day roosts on average every two to three days, they need to regularly explore new roosts (Fleischmann and Kerth 2014).

We provided one maternity colony with pairs of new bat boxes of the same design, differing only in their internal volume, fitted with RFID-loggers for continuous monitoring. This allowed us to control for other factors influencing roost choice and to record the nightly visits of RFID-tagged bats to the boxes prior to their occupation as day roosts. Finally, we measured the internal box temperature to rule it out as a confounding factor in roost choice.

We expected that bats would prefer boxes with a larger internal volume, indicated by a higher number of nightly visits and more frequent use as day roosts. Furthermore, we expected to find smaller roosting groups in boxes with a smaller volume. With regard to the internal temperature of the experimental boxes, we expected at most a small difference between box types, at least at night when bats are exploring and selecting their potential day roost (compare Tillman et al. 2021; Bakken et al. 2022). In summary, we hypothesize that boxes with larger internal volumes are more attractive to bats for two possible reasons: 1) because they can accommodate more individuals, thus facilitating social thermoregulation (Willis and Brigham 2007; Pretzlaff et al. 2010); and 2) because they may provide greater safety from predators (Sedgeley and O’Donnell 1999; Wendorf 2004).

## Material and methods

### Study site and study colony

We conducted this study in a deciduous forest close to the city of Würzburg (Northern Bavaria, Germany). Prior to the study period, 76 bat boxes of the type 2FN (Schwegler, Germany) were present in the roosting area (ca. 0.4 km^2^) of the study colony Guttenberg2 (GB2; see supplement Fig. S1a). We added a total of 26 brand new 2FN boxes (13 pairs) during the experiment. All boxes were located within the forest. Since 1996, the colony members have been marked in their first year of life with RFID-tags (Kerth et al. 2011). This has enabled long-term monitoring of the colony using RFID-loggers installed at the bat boxes used as day roosts (Kerth and König 1999; Kerth and Van Schaik 2012). During our study, the colony consisted of 63 adult females.

### Roost selection experiment

**Fig. 1:**
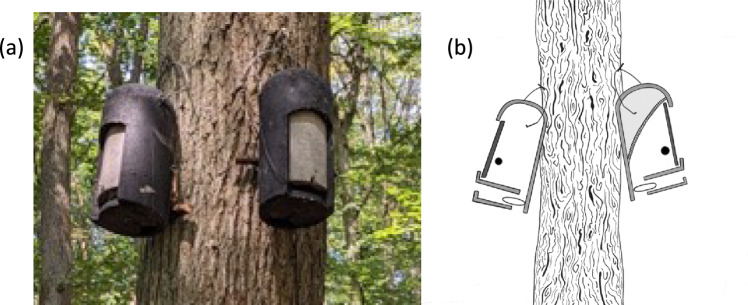
**a** Photo of an experimental box pair in our study site and **b** schematic representation of the experimental setup. Each pair consisted of a “control” box (here on the left) and a “reduced” box (right). The boxes are of black color and have two entrances, one at the front and one at the bottom. When the bats enter the box, the RFID-logger attached to the antenna (depicted by the loop) records the bats’ tags. In the “reduced” box, the upper part of the box is demarcated by kneaded concrete (gray striped bar). An iButton temperature logger was added to each box (black dot) at a height of ca. 7 cm from the inner floor

Each of the 13 experimental box pairs consisted of two boxes with different treatment types: “control” and “reduced” (Fig. 1). Both boxes are made of wood-concrete, have identical external dimensions (height: 36 cm, diameter: 16 cm, wall thickness: 3.5 cm) and are of black color (Fig. 1a). Boxes have two entrances, one rectangular in the front (7 cm × 2 cm) and one oval in the bottom of the box (6.5 cm × 2.5 cm). Both entrances provide access to an internal roosting chamber which has a steep inner floor 3 cm thick with a drop of 4 cm, giving a chamber height of 21 cm to 25 cm. The “reduced” box had a smaller internal roosting chamber with a maximum height of 15 cm while the “control” boxes were unmodified (Fig. 1b). The boxes were hung in pairs, in the understory on the same tree, approximately 25 cm apart. Both boxes faced southeast, similar to the other boxes in the area (Fig. 1a). The positions of the “control” and “reduced” boxes within an experimental pair (i.e., left or right) were chosen randomly for the first pair and were alternated for the following pairs. We permanently equipped each box with an RFID-logger to record the nightly visits of the bats and their use of the experimental boxes as day roosts. In each experimental box, regardless of the treatment, we placed a temperature logger (iButton; Thermochron, USA) inside the roosting chamber at a distance of ca. 7 cm from the inner floor.

In the “reduced” boxes, we reduced the available space for roosting by 50%. This calculation was done by filling a plastic bag with water inside the “control” and “reduced” box (2 vs 1 L). The dome of the box was enclosed by a laminated cardboard which was glued in place with silicone. To ensure that the bats could hold on the ceiling of the box, we applied an additional thin layer of kneading concrete. This material is similar to the wood cement that the box is made of. Additionally, we roughened the surface to provide a comparable texture. The space between the layer of kneaded concrete and the top of the box was not filled with material. The “control” boxes had a mass of 4.9 kg, while the “reduced” boxes weighed 5.1 kg, an increase of 4% in mass. To control for any aversion of the bats to the smell or texture of the materials added in the “reduced” box, we spread a small amount of the same materials on the wall of the “control” boxes.

### Experimental procedure

We conducted the experiment during the breeding season from 14-May to 20-Aug-2023. During this period, all bat boxes (26 experimental boxes and 76 normal boxes) were checked daily for the presence of bats without opening them by looking from the ground inside the box through the bottom entrance with a flashlight (Kerth et al. 2011). We downloaded the RFID-tag data on a laptop to identify the individuals that entered the boxes. At the beginning of the experiment, we hung up ten pairs of boxes, covering the main roosting area of the bats (see Fig. S1). If one box of an experimental pair was used as a day roost, the experiment was ended for this pair. When the bats left the occupied box, we removed the unused box type, thoroughly cleaned it, and re-set it up elsewhere with a new box of the complementary type as part of a new box pair. The occupied box type remained at the same location. In this way, we deployed a total of 13 pairs of boxes. The boxes used as day roosts remained in the same position as part of the regular monitoring program without temperature loggers and permanent RFID-monitoring. We programmed the temperature loggers placed in the experimental boxes to take a measure every 10 min as early as 15 May 2023 18:00 and as late as 20 August 2023 17:10 h (depending on when the box pair was deployed). We downloaded temperature data every two weeks.

### RFID data processing

The data collected during the RFID monitoring provided the bat identity, date and time of the record, and treatment type (“control” vs “reduced”). We conducted data processing, analyses, and visualization using R v.4.3.1 (R Core Team 2023). For data parsing and wrangling we used the packages stringr (Wickham and RStudio 2019), stringi (Gagolewski 2022), lubridate (Grolemund and Wickham 2011) and dplyr (Wickham et al. 2021). Statistical tests were computed using exactRankTests (Hothorn and Hornik 2022). For data visualization, we used the python libraries matplotlib (Hunter 2007) and seaborn (Waskom 2021).

We analyzed the RFID-logger data, chronologically sorted, and derived the following variables according to Hernández-Montero et al. (2020): treatment, visit, experience of the bat with a given box (experienced or naive), information type (social or non-social), pair discovery, and day roost use. The algorithm for data processing is depicted in Fig. S2 in the supplement. The variables were defined as follows:

**Treatment**: defines the treatment of a given box: “control” or “reduced”. “Control” boxes represent an unmodified box; “reduced” boxes have a reduced internal volume.

**Visit**: is defined as the first record (box entry) of a bat at a given box during a given night. Only the first visit of each individual was considered to avoid pseudo-replication due to revisits to the same box during the night. Each visit had a date-time stamp and an associated ID.

**Experience:** a bat was considered naïve when it visited a given box for the very first time. Otherwise, the bat was considered to be experienced with regard to this box.

**Information type**: visits were categorized into “social” and “non-social” depending on whether information about a given box could have been provided by conspecifics. Non-social visits were assigned when a bat visited (entered) a box alone or was the first to be recorded in a group of naïve bats. Individuals were considered to have social information available when they visited a given box together with an experienced individual. If the time difference between the records of different bats was less than 60 s, bats were considered to be visiting the box together, therefore, potentially using social information. A time span of 60 s was selected according to Hernández-Montero et al. (2020). To inspect the consistency of results, we also performed statistical analyses using an 180 s time span according to Kerth and Reckardt (2003; see supplement Figs. S3–S5).

**Pair discovery**: is defined as the very first visit of a bat to a given experimental pair regardless of the treatment type of the box. Therefore, a pair discovery can occur by visiting a “control” or a “reduced” box.

**Day roost use**: if bats are roosting inside a given box during the day, this box is defined as a day roost. RFID records at a given box that was used as a day roost are not counted as visits because bats tend to return to their day roost during the night and on consecutive days and already have selected it as a roost before.

We excluded from the analysis the juveniles tagged in August because they might still be dependent on their mothers for selecting roosting sites, and because they were tagged by the end of the experiment.

### Data analysis

We investigated the influence of the internal box volume on roost selection by analyzing the explorative and roosting behavior of the bats. Explorative behaviors included the discovery of experimental pairs and subsequent visits to experimental boxes according to our definitions. Since roost temperature can influence roost choice (Kerth et al. 2001), we also analyzed whether roost temperatures differed between treatments (“control” vs. “reduced”).

Before analyzing the explorative behavior of the bats, we investigated whether the bats could discriminate the condition of the box before entering it. For this purpose, we carried out two binomial tests with an expected proportion of 0.5. The first binomial test compares the proportion of “control” and “reduced” boxes visited during the very first visit recorded for each of the 13 experimental box pairs. The second test compares the proportion of visits between treatment types for all individual bats that used non-social information according to our definition on their very first visit to an experimental box. We considered only non-social visits to reduce the possibility that social information influenced an individual’s roost choice, which would lead to non-independent individual data.

We analyzed the explorative behavior, which is indicative of roost selection by Bechstein’s bats by comparing the number of pair discoveries and visits. In assessing for differences between treatments, we analyzed pair discoveries and visits using pooled information data (social and non-social combined). Additionally, we compared the differences in the use of social and non-social information for both treatment types. To do this, pair discoveries and visits were aggregated according to the information type (social vs. non-social). We computed statistical comparisons between treatments using the Wilcoxon test. We addressed normality and homoscedasticity (*p* < 0.05) using Shapiro–Wilk and Bartlett tests respectively.

Furthermore, we compared the ratio of the two information types (social vs non-social) used when visiting an experimental box between both treatments (“control” vs “reduced”). Finally, we compared the number of days that bats used “control” and “reduced” boxes as a day roost as this may indicate a preference for one or the other treatment type. The total number of boxes used as a day roost was compared between treatments using a binomial test with an expected proportion of 0.5. For each treatment type, we summarized the minimum, maximum, and mean number of days that bats used a box as day roost. Finally, we also summarized the number of bats roosting together in a box (group size) for each treatment type.

### Internal temperature analysis

To analyze the temperature data, we merged all files collected from the iButton temperature loggers. The resulting data frame included the datetime, batdate, hour in which the record falls, temperature, pair number and treatment. A batdate encompasses the nightly activity time of the bats into a single date, starting at 07:00 and ending at 06:59 of the following day. To avoid artifacts, we excluded from the analysis the measurements taken when the boxes were opened to collect data or to capture the bats. We also excluded data from box pairs where there were no records for both box types on a given day due to a full memory of one of the iButtons, to avoid unbalanced comparisons.

To assess differences between box types, we calculate the mean temperature for each timestamp per box type, and then visually compare the 24 h detrended temperature pattern between box types. Detrended temperatures were calculated based on the substraction of linear least-squares regression from the mean temperature using the “detrend” function of the time series analysis module of the statsmodels (v0.14.4) python library (Seabold and Perktold 2010). To simplify the visualisation, only the temperatures recorded in July 2023 are shown; the full dataset is presented in the supplement, Fig. S6.

In addition, we examine a subset of the temperature data corresponding to the warmest day by selecting the batdate with the maximum temperature recorded inside the boxes. We chose the warmest day for our analysis, following the approach of Tillman et al. (2021), as we expected to see clearer differences between the box types under extreme conditions. Having selected the warmest batdate, we resampled the temperature data on a hourly basis (6 measurements per hour) and calculated the mean, standard deviation (SD), across all experimental boxes with data available for the selected batdate. We also calculate the hourly average and range of temperature difference by subtracting the temperature of the “reduced” boxes from that of the “control” boxes within each experimental pair (i.e., control—reduced).

We assessed internal temperature differences between treatments in two ways. First, visually by plotting the hourly mean and error bars using ± 1.96 SD, as well as the hourly mean and range (minimum and maximum) difference between box types, the same visualization was done for each pair (see Fig. S7). Second, we compared the overall mean internal temperature recorded during the night between box types using a two-tailed paired t-test after assessing for normality and homoscedasticity using the Kolmorogov-Smirnov (*p* = 0.99) and Levene’s tests (*p* = 0.91), respectively. For this second test, we pooled the data from all boxes of the same treatment type during the night between 21:00 and 06:00. This time window covers the activity period of the bats during the night when they are likely to be looking for new roosts.

## Results

**Fig. 2:**
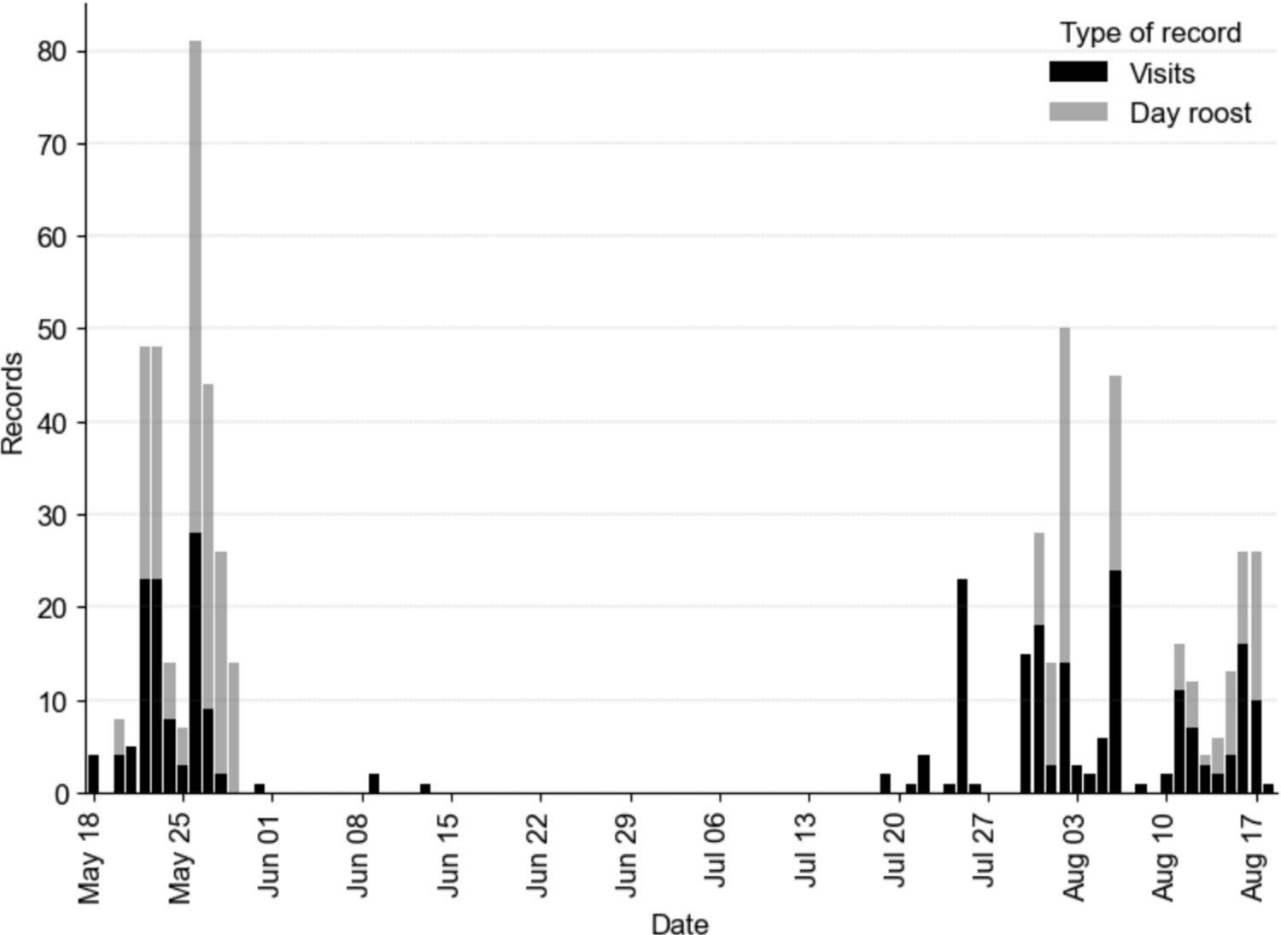
Number of records at experimental boxes over the course of the experiment. Black bars show the number of nightly visits to experimental boxes; grey bars represent the number of bats using one or more experimental boxes as a day roost

Over the 99-day study period, bats entered experimental boxes on 38 days, either by visiting them at night or using them as day roost. In total, 61 adult female bats were recorded at experimental boxes. All 13 experimental box pairs were visited at least once. Bat activity at experimental boxes (visits and use as day roosts) showed two peaks, one before and one after the lactation period, separated by a period of inactivity *i.e.* the bats did not visit any experimental box, but were present in the study area roosting in non-experimental boxes (Fig. 2). The highest activity was recorded between the middle of May and the beginning of June in the weeks before the birth of the young which occurred around the 15th of June. The second period of activity began at the end of July, at the end of the lactation period, and lasted until the end of the experiment.

### Pair discoveries

Of the 13 pairs of boxes, 10 were discovered by visiting the “control” box first and three by visiting the “reduced” box (binomial test: *p* = 0.09). Of the 36 individuals that used non-social information during their first visit to an experimental pair, 31 individuals visited the “control” box and 5 visited the “reduced” box (binomial test: *p* < 0.001).

**Fig. 3:**
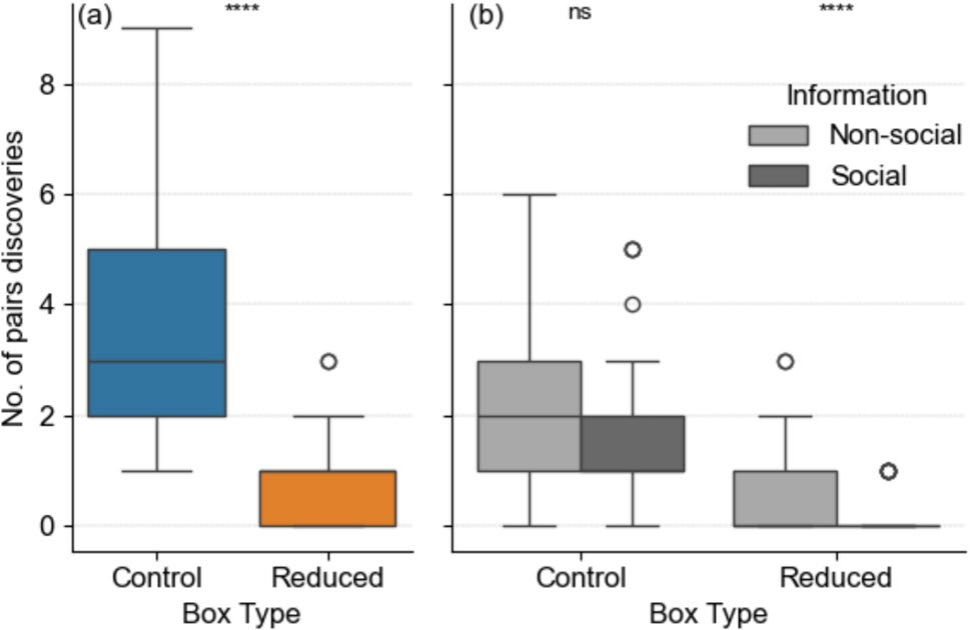
Box plots of **a** the number of pair discoveries to the experimental box pairs of Bechstein’s bat (n = 61) for the two treatment types, “control” and “reduced”, and **b** the number of pairs discovered performed by Bechstein’s bats (n = 61) using non-social and social information per treatment type (“control” vs “reduced”). Results from the Wilcoxon signed-rank test are shown as: ns *p* > 0.05; **** *p* < 0.0001

Overall (pooled social and non-social information data), the 61 bats discovered more box pairs by visiting the “control” box first (Fig. 3a, Wilcoxon, W = 3459.5, *p* < 0.0001, n = 61). With regard to the information types being used (social vs non-social), there was no significant difference for the “control” boxes (Fig. 3b, Wilcoxon, W = 2057, *p* = 0.2985, n = 61). However, “reduced” boxes were discovered more often using non-social information (Wilcoxon, W = 2616.5, *p* < 0.0001, n = 61).

### Visits

**Fig. 4:**
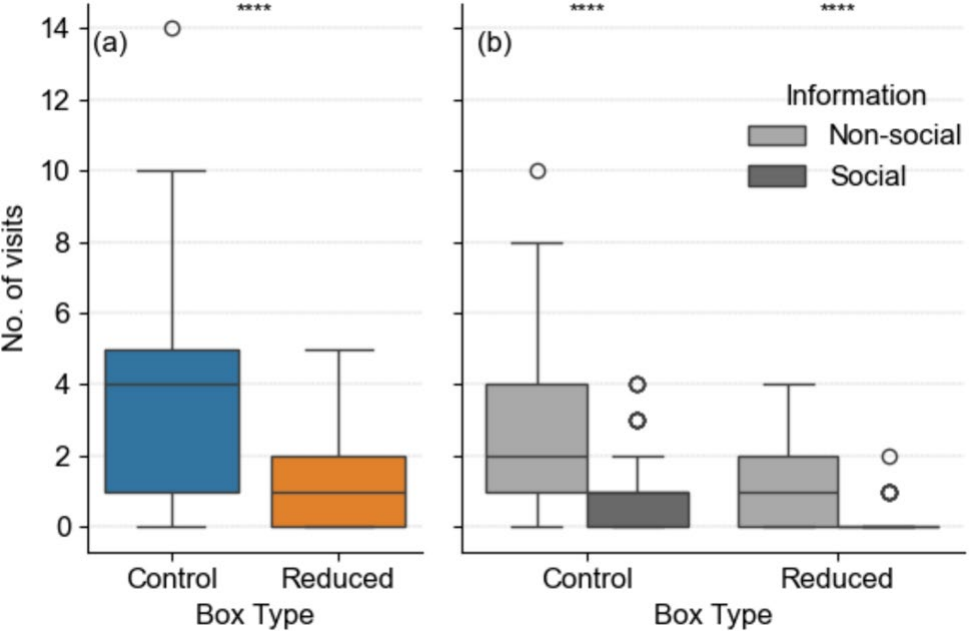
Box plots of **a** the number of visits of Bechstein’s bats (n = 61) to experimental box pairs for the two treatment types, “control” and “reduced”, and **b** the number of visits by Bechstein’s bats (n = 61) using non-social and social information per treatment type. Results from the Wilcoxon signed-rank test are shown as: ****p < 0.0001

“Control” boxes were visited significantly more often than “reduced” boxes: 218 visits from 55 individuals were recorded at “control” boxes (76%) and 69 visits from 34 individuals at “reduced” boxes (24%; Fig. 4a, Wilcoxon, W = 0.89538, *p* < 0.0001, n = 61).

At both box types, we recorded significantly more non-social than social visits (Fig. 4b, “control” Wilcoxon, W = 2662.5, *p* < 0.0001, n = 61; “reduced” Wilcoxon, W = 2648, *p* < 0.0001, n = 61). However, the ratio of non-social to social visits differed between treatments. The “control” boxes show a ratio of 72% non-social to 28% social visits, whereas the “reduced” boxes had 87% non-social to 13% social visits. Thus, while there were more non-social visits in both cases, the proportion of non-social visits was significantly higher in boxes with a reduced internal volume (Fisher exact test: *p* = 0.015).

### Day roost use

A total of 12 boxes were used as day roosts, of which 10 were “control” and two were “reduced” boxes (Fisher exact test: *p* = 0.005). In total, experimental boxes were used as day roosts on 20 days. On average, the 10 occupied “control” boxes were used for 2.5 days (range 1–9 days); while the two occupied “reduced” boxes were used as a day roost for one day. Of the 61 individuals, 60 used the experimental boxes as day roosts at least once, all of which were roosting in “control” boxes, and only 6 individuals in “reduced” boxes. The average group size was 12.5 individuals for the “control” boxes (range 2–31 bats) and 3.0 (range 2–4 bats) for the “reduced” boxes.

### Internal temperature analysis

**Fig. 5:**
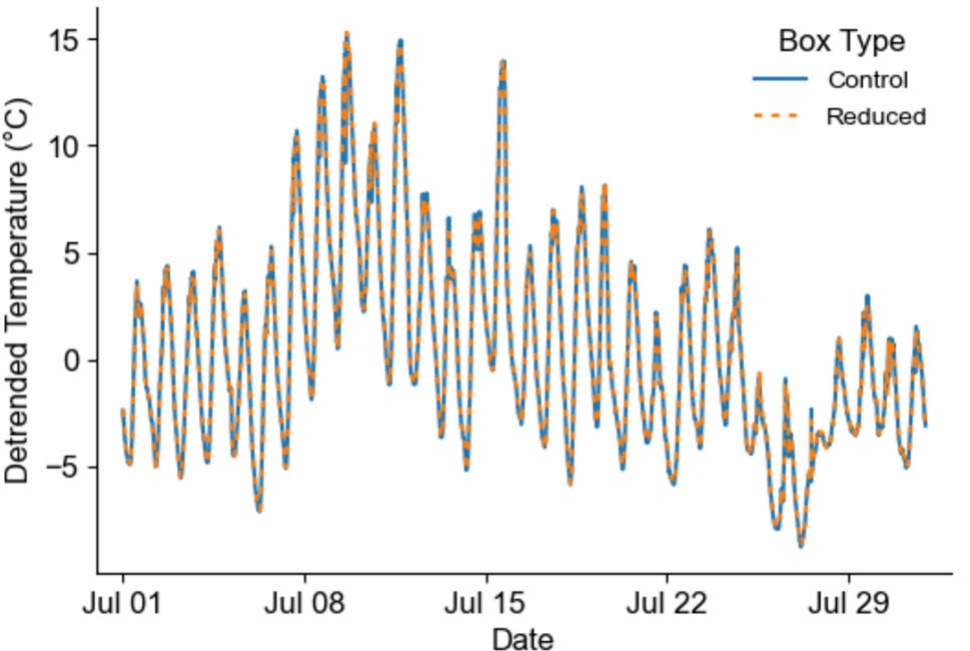
Detrended temperature recorded in July 2023 for control (blue line) and reduced (orange dashed line) boxes. The 24 h detrended temperature was calculated based on the residuals of the linear regression of the mean temperature for each datetime per box type. The detrended mean temperature recorded in each box type follows the same pattern

**Fig. 6:**
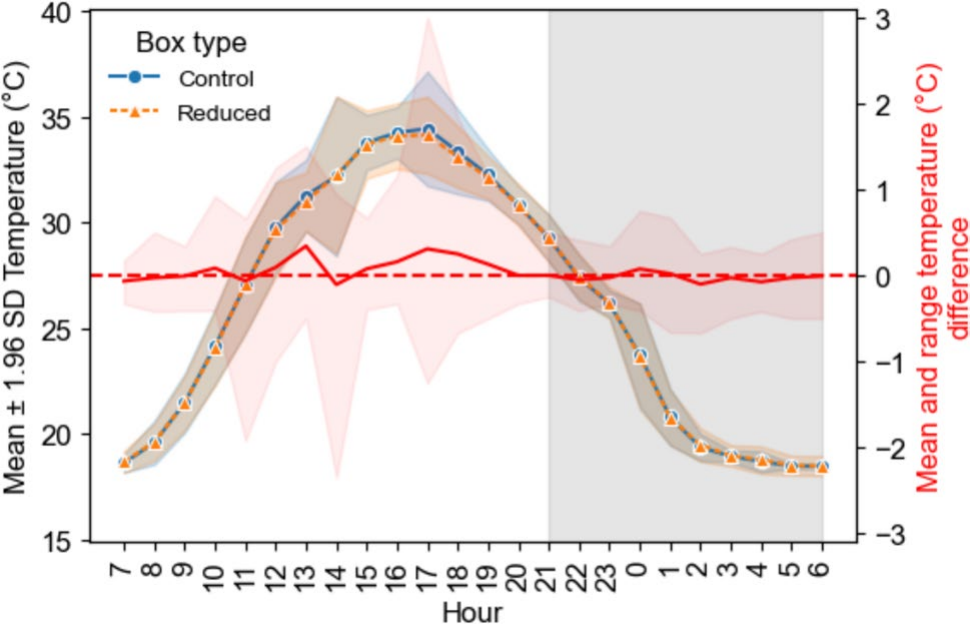
Hourly mean ± 1.96 SD temperature of the control (circles) and reduced (triangles) boxes on 2023-07-11 (n = 9 experimental pairs). Red line represents the hourly mean and range difference between box types. Note that hours between 07:00 and 06:00 of the following day represent a day of activity for bats. The gray shaded area represents night hours when bats are more likely to explore for roosts

Visual inspection of the 24 h detrended temperature showed an identical pattern between “control” and “reduced” boxes (Fig. 5; supplement Fig. S6). The warmest date of bat activity (*i.e.* batdate) according to the maximum temperature recorded at the experimental boxes was 2023–07-11. Both box types (n = 9 pairs) showed a similar temperature pattern, with temperature differences ranging within 3 °C over the whole day. However, the differences between the box types narrowed to less than ± 1 ºC during the night between 21:00 and 06:00 (Fig. 6). This pattern was also observed for each of the 9 pairs analyzed on this date (Fig. S7). We found no significant difference in the mean internal temperature between box types at night when bats are active and searching for roosts (t-test = -1.61, df = 539, *p* = 0.10).

## Discussion

Our field experiment shows that female Bechstein’s bats can base their roost choice on the space available for roosting. By presenting pairs of boxes that differed only in their internal volume, we were able to largely exclude potentially confounding factors for the bats’ roost selection, such as the locality of the boxes in the forest and box design (shape, external size, color and material). As the temperatures inside the boxes with different volumes did not differ, roost temperature could be excluded as a confounding factor in our experiment.

### Bat activity

The records of bats at experimental boxes showed two phases of increased activity, separated by a period of inactivity in June and July, which can be explained by the females’ reproductive phenology. Bechstein’s bats give birth to their young mid-June (Mundinger et al. 2021), as also observed in our study. During the 4–6 weeks of lactation, females change their roosts less frequently (personal observations) and consequently explore new roosts less frequently. This does not mean that bats were not present in the study area, they were present in other previously installed (non-experimental) bat boxes but did not visit the experimental boxes during this time. Non-experimental boxes present in the study area have an identical volume of the control boxes from our experiment.

### Can bats recognize the available space for roosting before entering the box?

We had first investigated whether bats can detect the available space in a box before entering it. We found that for the 13 discovered box pairs the first arriving bat entered in 10 cases the “control” box first and in 3 cases the “reduced” box (*p* = 0.09). When we analyzed the first non-social visit of individual bats to the boxes, we found that 31 out of the 36 bats entered the “control” box first (*p* < 0.001). Taken together, our analyses suggest that the bats were able to assess —by unknown means— the amount of space available for roosting in a box before actually entering it. This was unexpected as in similar pairwise roost choice experiments, testing different box characteristics, female Bechstein’s bats needed to enter boxes in order to assess whether they were suitable for roosting (Kerth and Reckardt 2003; Hernández-Montero et al. 2020).

Our finding raises the question, how the bats were able to discriminate between “control” and “reduced” boxes before entering them? Since the same box type (2FN) was always used in our experiment, all boxes had the same size and design without any apparent external cue indicating the box’s internal volume (Fig. 1). In addition, to the extent possible, we controlled for differences in the odor potentially caused by the materials used to limit the roosting space in the “reduced” boxes by adding the same materials to the “control” boxes. In this context, it is important to note that olfactory cues do not seem to play an important role in the roost selection of bats (Ruczyński et al. 2007; Brown and Carter 2022). We therefore consider it unlikely that the bats used odor cues to differentiate between the two box types in our experiment. Currently, it remains an open question how Bechstein’s bats were able to recognize reduced boxes before entering them.

### Bat box volume did not influence roost temperatures

When comparing the 24 h detrended temperature profile between box types, it is clear that they follow the same pattern despite the use of an independent linear model for each box type and sampling day of bat activity (Fig. 5). When we focused our analysis on the warmest day, the visual inspection of the hourly mean temperature differences (Fig. 6; supplement Fig. S7), shows that the absolute difference between box types was < 1 °C at night. Futhermore, none of the box types were consistently warmer or cooler during the night. This was also supported by our two-tailed t-test, which showed no significant differences between box types (*p* = 0.10). Very similar temperature profiles were also observed between the two box types throughout the study period (see Fig. S6).

Although we only used one temperature logger per box to measure temperature, we are confident that this is sufficient to measure the internal temperature of the 2FN boxes. Previous internal temperature data collected using two temperature loggers, one installed at the top and one at the bottom of the internal chamber, showed no differences between the two loggers, particularly at night (mean ± SE: top: 21 ± 0.21 ºC; bottom: 20.9 ± 0.19 ºC, n = 21 boxes; Janis Wolf unpublished data). This lack of a thermal gradient in the boxes could be due to the size of the roosting chamber of the 2FN bat boxes, the material from which they are made, the shaded placement of the boxes, and the small entrance that could prevent high airflow. For comparison, Tillman et al. (2021) investigated the thermal gradient in different versions of wooden rocket type bat boxes deployed in open conditions, with a height of 94 cm and a wide entrance that allows for air flow. Despite the very different box design compared to the 2FN boxes, their study showed that several versions of the rocket box also had no thermal gradient between 00:00 and 06:00 when bats are likely to be searching for roosts. A follow-up study, using the same type of rocket boxes, confirms that temperature differences between different heights in the box are rather small at night (< 3 °C) and not monotonic (Bakken et al. 2022). In our study, the temperature differences are even smaller (< 1 °C), therefore we assume that our box modification was too subtle to significantly alter the internal temperature of the reduced boxes. With these results, we discard the internal temperature of the boxes as a confounding factor and conclude that the box types differed only in their internal volume.

### Evidence for a direct influence of the boxes’ internal volumes on roost choice

Our hypothesis that bats prefer boxes with a larger volume was confirmed by a three times higher number of visits to “control” boxes than to “reduced” boxes (218 vs. 69). It has been previously shown that female Bechstein’s bats visit suitable roosts more often than unsuitable roosts before using them as day roosts (Kerth et al. 2006). Therefore, we conclude that a lower number of visits at “reduced” boxes indicates that the boxes with a smaller internal volume are considered less suitable by the bats. While bats visited boxes of both types more frequently using non-social information (non-social visits), the frequency of non-social visits was higher for “reduced” than for “control” boxes (87% versus 72%). During their nightly roost exploration, female Bechstein’s bats transfer information about new suitable roosts between colony members through a leading-following behavior, visible in the social visits (Kerth and Reckardt 2003; Fleischmann and Kerth 2014; Hernández-Montero et al. 2020). The significantly lower number of social visits at “reduced” boxes therefore suggests that bats recruited fewer colony members to “reduced” boxes than to “control” boxes. We conclude from this that the bats perceived “reduced” boxes as unsuitable already during the exploration process.

Ultimately, the preference for boxes with a larger volume was reflected in their more frequent use as day roosts (Fisher’s exact test, *p* = 0.005). To the best of our knowledge, no other field experiments have directly examined the influence of the available space on roost selection in bats. In contrast, the importance of available space in the selection of nesting sites is well documented in eusocial insects. For example, it has been shown that ants are able to estimate the volume of cavities and prefer larger nesting sites that do not limit the growth of the colony (Thomas 2002; Katzerke et al. 2010). Honeybees also consider the volume of a cavity when colonies need to find a new nesting site during swarming, with the preferred volume generally depending on the size of the swarm (Seeley 1977; Villa 2004).

### Why are larger roosting spaces preferred?

As expected, we found larger groups of bats roosting in “control” boxes. The average group size in “control” boxes was four times larger than that of “reduced” boxes (12.5 vs. 3). It is well-documented that communal roosting provides bats with energetic benefits through social thermoregulation. Thus, choosing a roost large enough to allow for thermoregulatory benefits of communal roosting is crucial for many bat species (Kunz 1982; Willis et al. 2006; Willis and Brigham 2007). For example, *Eptesicus fuscus*, *Plecotus auritus,* and *Myotis nattereri* formed larger groups in roosts with a larger internal volume (Willis et al. 2006; Dodds and Bilston 2013). A previous study of female Bechstein’s bats roosting in bat boxes identical to our “control” boxes, found that individuals belonging to roosting group of less than 10 individuals had a 15% higher metabolic expenditure compared to individuals roosting in groups larger than 10 individuals (Pretzlaf et al. 2010). While the benefits of social thermoregulation can explain the observed preference for boxes with a larger volume, the question remains why “reduced” boxes were used at all as day roosts. The two “reduced” boxes used as day roosts were occupied after the lactation period. Larger group sizes can provide an energetic advantage if bats are homeothermic; however, group size is less important when bats enter torpor to minimize their energetic expenditure (Pretzlaff et al. 2010). Females enter torpor less frequently during gestation and lactation than after weaning their offspring (Lausen and Barclay 2003; Dietz and Kalko 2006; Solick and Barclay 2006; Pretzlaff et al. 2010; Bergeson et al. 2021). The reduced need for social thermoregulation after the lactation period may therefore explain why two “reduced” boxes were used as day roosts at the end of the experiment in August.

While the results of our experiment are consistent with Bechstein’s bats preferring a larger roost to benefit from social thermoregulation, this is not the only possible explanation for why “reduced” boxes were largely avoided as day roosts. Bats roosting in a “reduced” box were closer to the box entrance compared to bats roosting in a “control” box. Roosts that require bats to roost near the entrance have been shown to provide less protection from predators (Sedgeley and O’Donnell 1999; Wendorf 2004). This may explain, why common pipistrelles (*Pipistrellus pipistrellus*) and common noctules (*Nyctalus noctula*) use woodpecker cavities only after they have reached a certain height above the entrance due to decay (Stratmann 2007). Thus, “reduced” boxes may have been perceived by Bechstein’s bats as less secure than “control” boxes.

### Implications for forest bat conservation

Although our results are based on Bechstein’s bats only, other tree-dwelling bat species with similar body mass (ca. 8–12 g) and colony sizes (10–80 individuals) might show the same roosting requirements, e.g., Natterer’s bats and brown long-eared bats. However, for species that use larger cavities or crevicies roosting space may play a minor role. Our study underlines the importance of providing forest-dwelling bats with suitable roosts when deploying bat boxes (compare Crawford and O’Keefe 2024). The results of our field experiment suggest that it is important that roosts provide enough space for bats to form groups that allow for social thermoregulation and safe roosting. This should be considered not only in box supplementation programs but also in the creation of artificial tree cavities also known as forest aging (Goldingay and Stevens 2009).

In many managed forests, often only relatively young trees remain as potential roosts for bats, and old or dead trees with large diameters are much rarer than in natural un-managed forest (Mickleburgh et al. 2002; Frick et al. 2020). However, due to their smaller diameters, young trees could provide insufficient space for large groups of bats as well as a cooler microclimate due to their lower thermal mass. Consequently, bats need to roost in smaller groups, which could result in disadvantages in social thermoregulation (Willis et al. 2006; Willis and Brigham 2007) and possibly higher predation rates (Sedgeley and O’Donnell 1999; Wendorf 2004). Alternatively, bats could avoid these roosts, which would significantly reduce the number of roosting opportunities. Either way, this could have a detrimental effect on tree-dwelling bat populations. While it remains to be investigated whether bats avoid tree-cavities that are too large, our results underline the importance of natural roosts that are spacious enough to host larger groups of bats and/or to roost safely. Moreover, if the deployment of artificial roosts is necessary to compensate for the lack of natural roosts in young forests it is important that the used bat boxes meet the requirements of the target species, as different bat species are known to have different roosting requirements.

## Supplementary Information

Below is the link to the electronic supplementary material.

Supplementary file1 (DOCX 5650 KB)

## Data Availability

Datasets to conduct data analysis are available on OSF at https://osf.io/9us32/?view_only=4463c08ddef94edfb77e4d2f275d23e4.

## Code availability

Code for the temperature analysis is available on OSF at https://osf.io/9us32/?view_only=4463c08ddef94edfb77e4d2f275d23e4 as an html report.
