# Supplementary material for: Field experiment reveals that female Bechstein’s bats (Myotis bechsteinii) select bat boxes based on the space available for roosting
Source: Oecologia. 2025 Apr 2;207(4):58. doi: 10.1007/s00442-025-05700-9 (PMC11965151; doi:10.1007/s00442-025-05700-9)

**Electronic Supplementary Material**

**Field experiment reveals that female Bechstein’s bats (*Myotis bechsteinii*) select day roosts based on the space available for roosting**

Willemsens, Christina; Kerth, Gerald; Hernández-Montero, Jesús

Zoological Institute and Museum, Applied Zoology and Nature Conservation, Greifswald University, Greifswald, Germany.

**b)**

**a)**


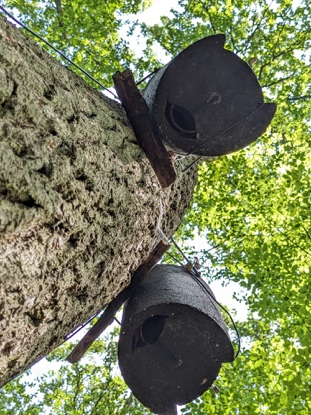

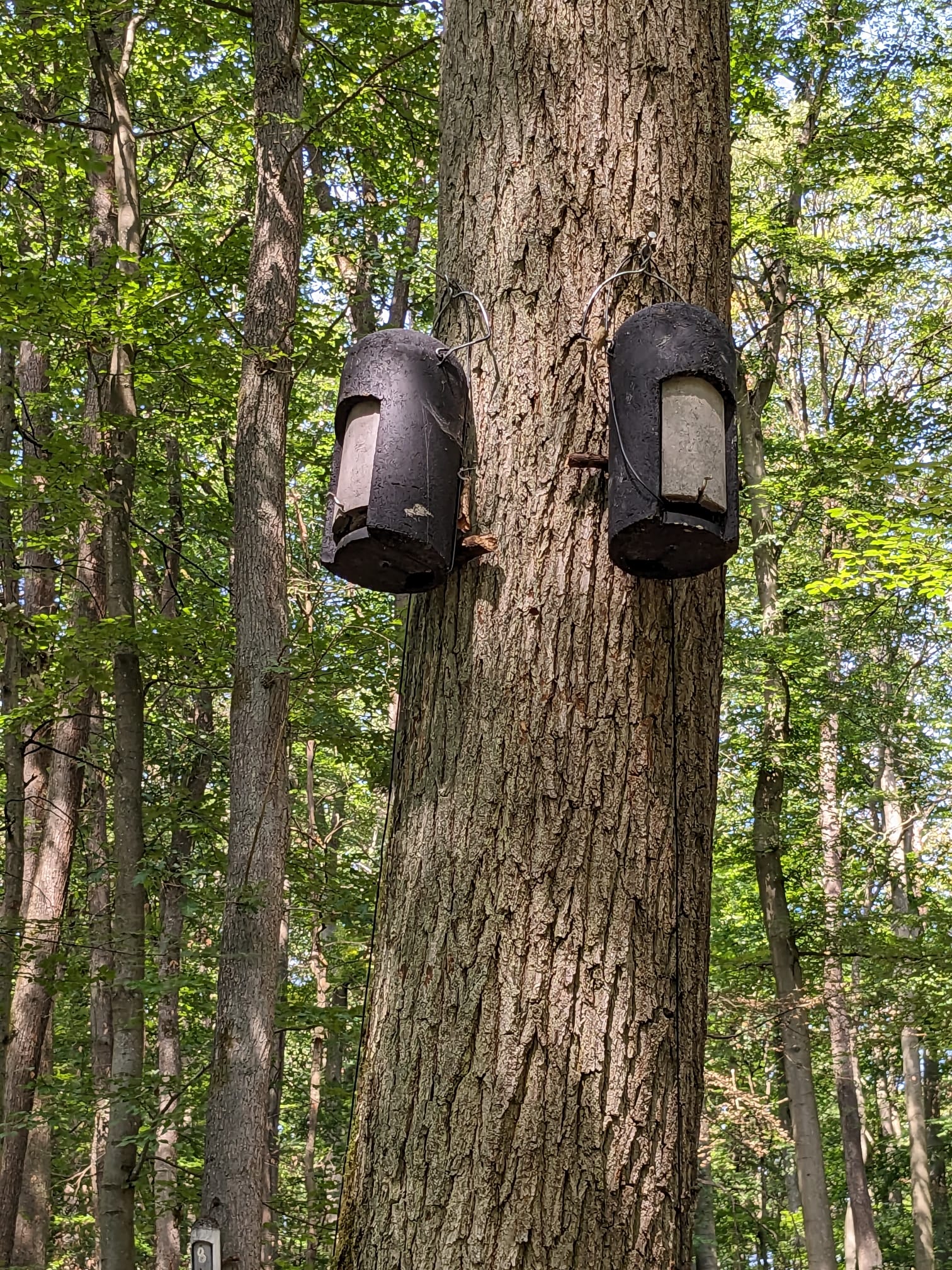

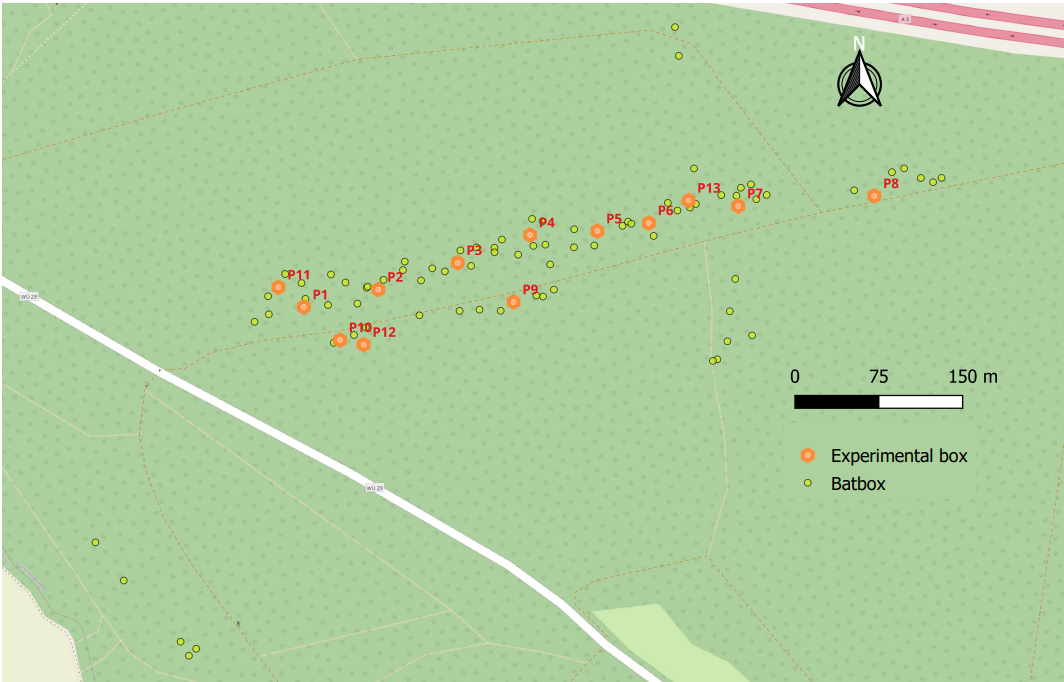


**c)**

**Fig. S1:** a) Map of the study site. There were 76 boxes (yellow dots) distributed in the area as part of the general monitoring. During the experiment, a total of 13 box pairs (orange hexagon; numbers refer to the corresponding box pair) were added. b) Experimental pair consisting of a unmodified “Control” box and a “Reduced” box, boxes have the same external dimensions. c) Detail of the bottom entrance of the boxes, the logger antenna can be appreciated from below.


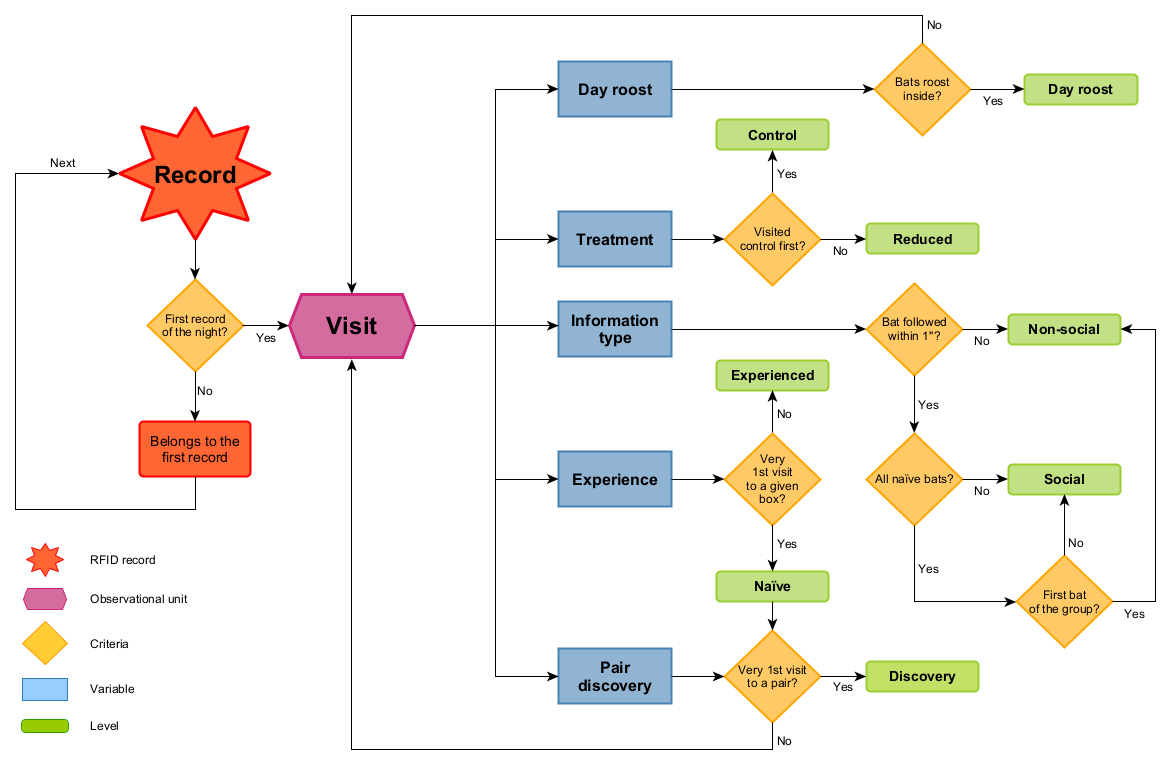


**Fig. S2:** Algorithm used for processing raw RFID data retrieved from the experiment (based on Hernández-Montero et al., 2020). The diagram shows the variables and their corresponding levels assigned to each visit according to the processing criteria.

**Results based on an alternative timespan of 180sec**


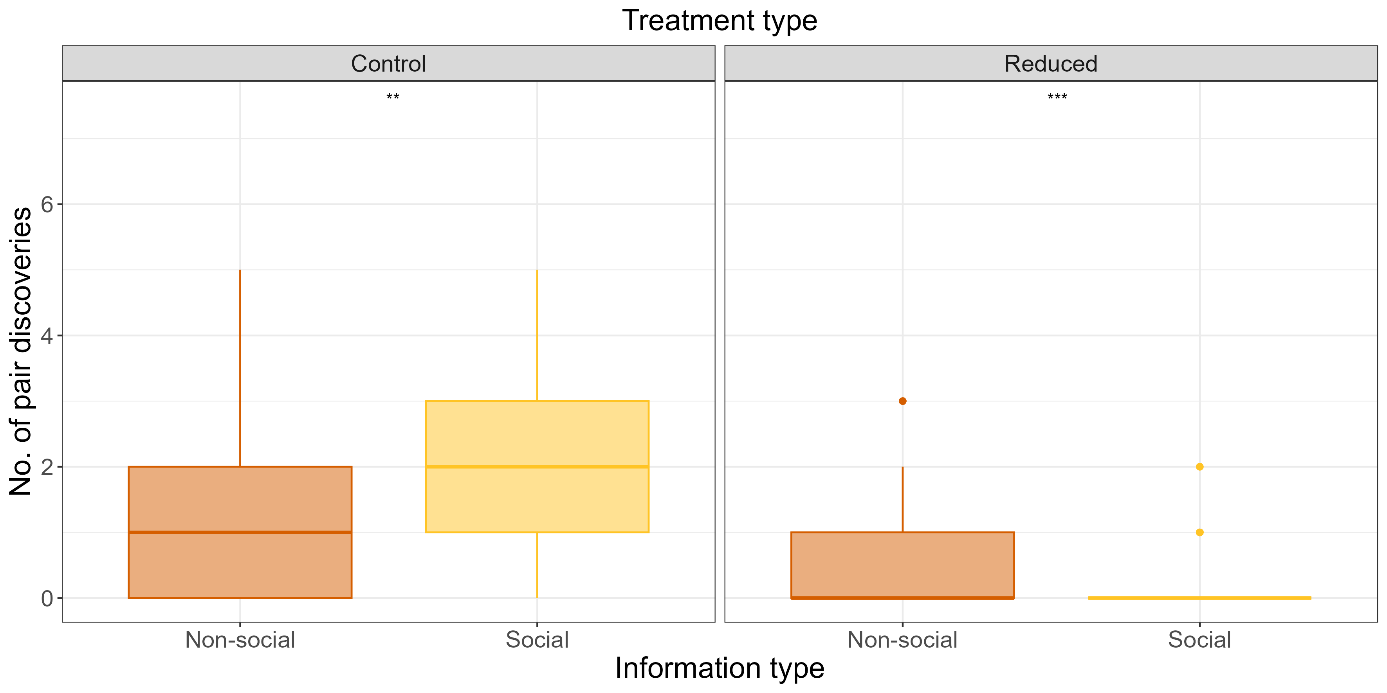


**Fig. S3:** Box plot of the number of pairs discovered performed by Bechstein’s bats (n = 61) using non-social and social information per treatment type (“Control” vs “Reduced”). Results from Wilcoxon signed-rank test shown as: ** p > 0.01; *** p < 0.001. Based on a timespan of 180 seconds.


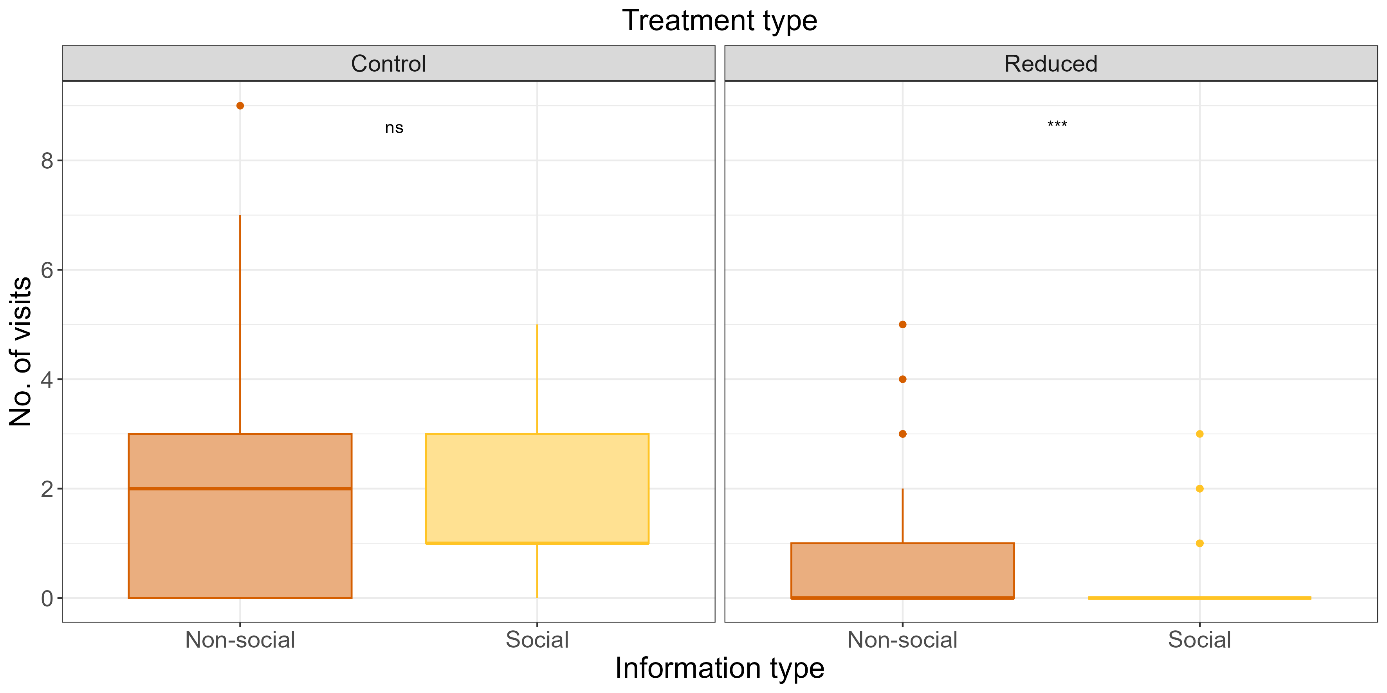


**Fig. S4:** Box plot of the number of visits by Bechstein’s bats (n = 61) using non-social and social information per treatment type. Results from Wilcoxon signed-rank test shown as: ns p > 0.01; ******* p < 0.001. Based on a timespan of 180 seconds.


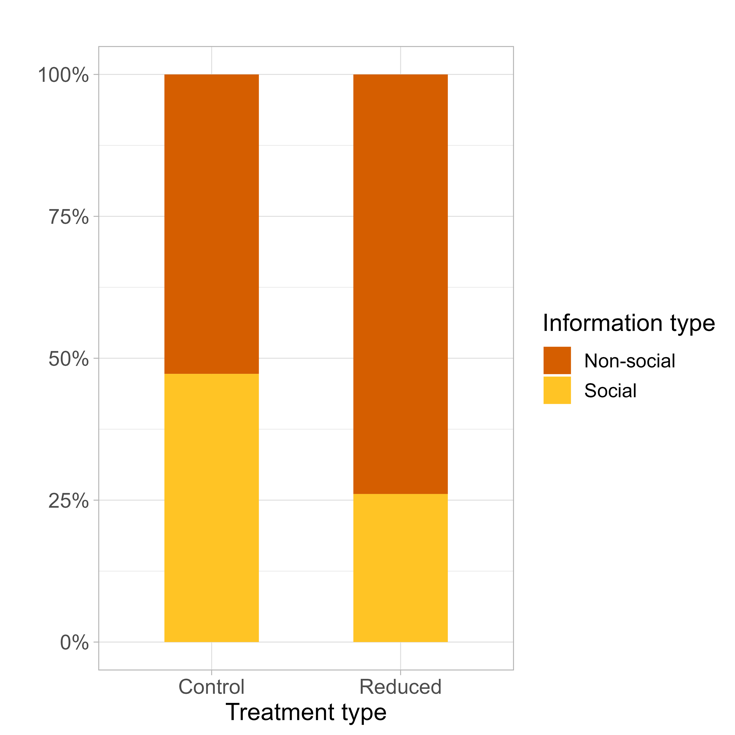


**Fig. S5:** Comparison of the ratio between the two information types (social vs non-social) used when visiting the “Control” and “Reduced” boxes. Results based on a timespan of 180 seconds.

**Fig. S6** Detrended temperature of Control and Reduced boxes for the study period. The 24h detrended temperature was calculated based on the residuals of the linear regression of the mean temperature for each datetime per box type.

**
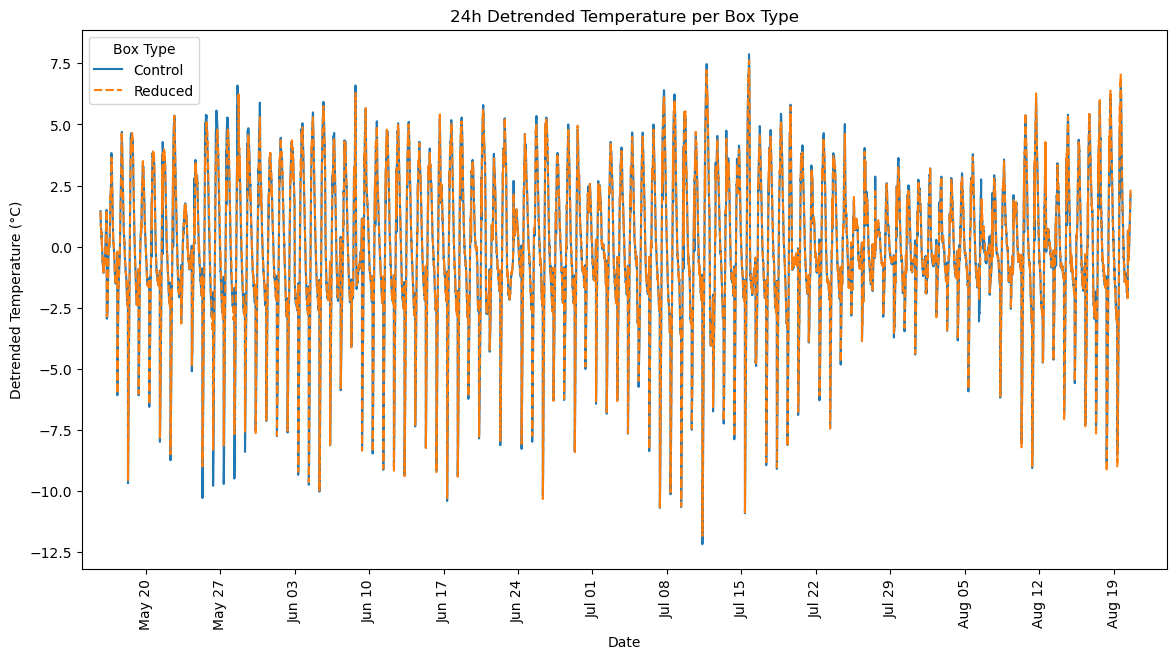
**

**Fig S7** Hourly mean temperature of the Control (-•-) and Reduced (-x-) box belonging to the experimental pairs recorded on 2023-07-11. Shaded ribbons represent ± 1.96 SD. Red line represents the hourly mean difference and range (min - max) between box types. Note that hours between 07:00 and 06:00 of the following day represent a day of activity for bats. The gray shaded area represents night hours when bats are more likely to explore for roosts.


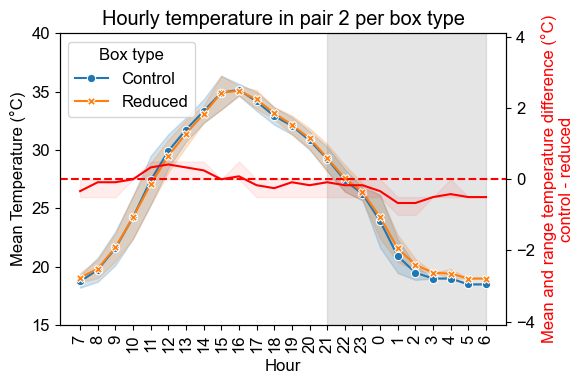

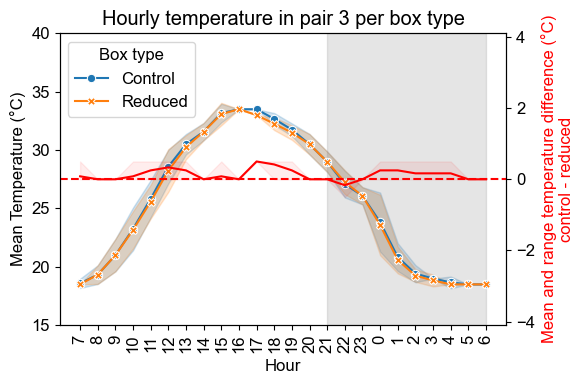


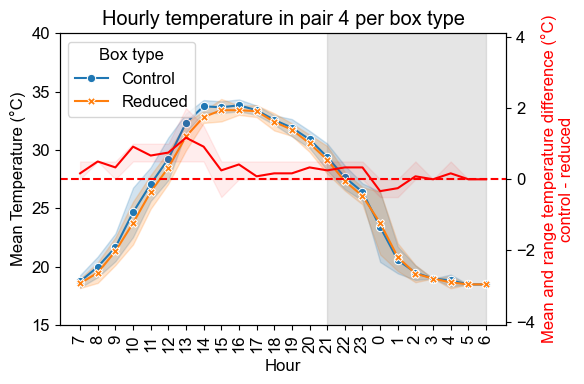

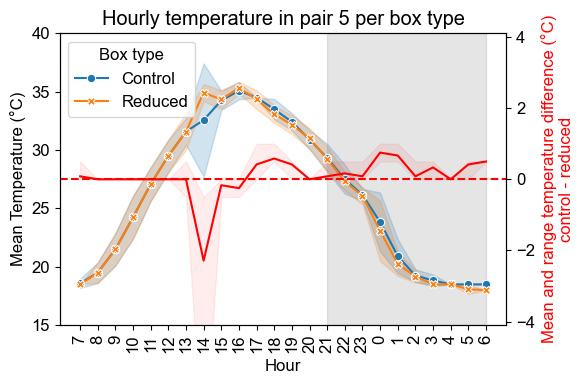


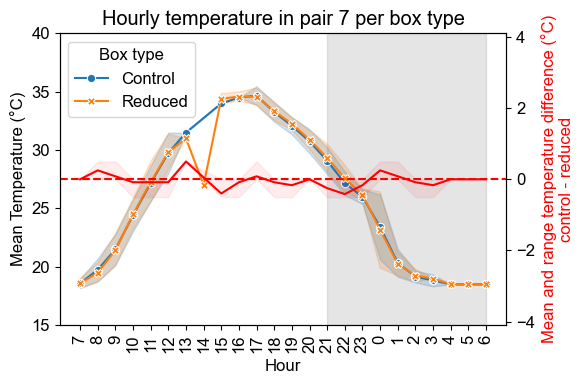

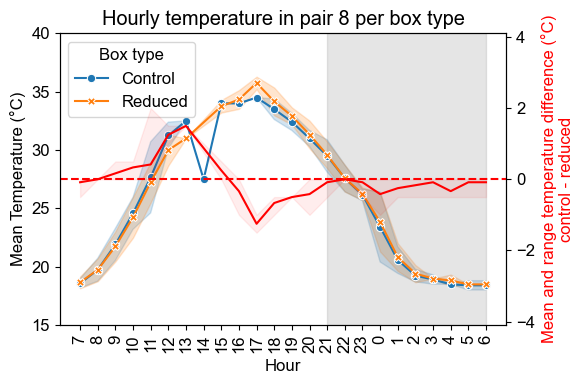


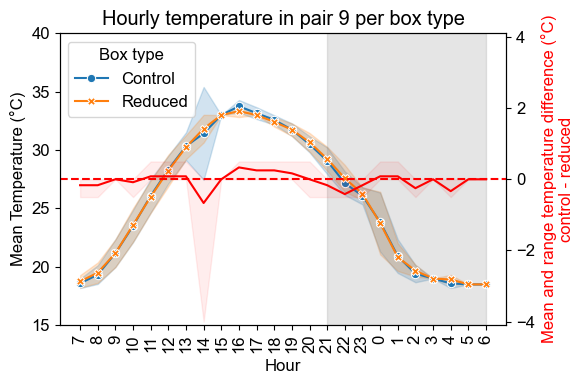

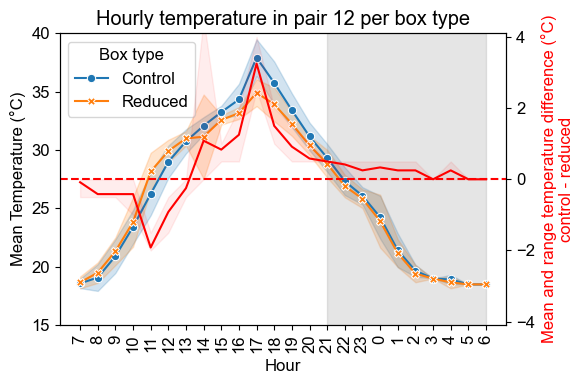


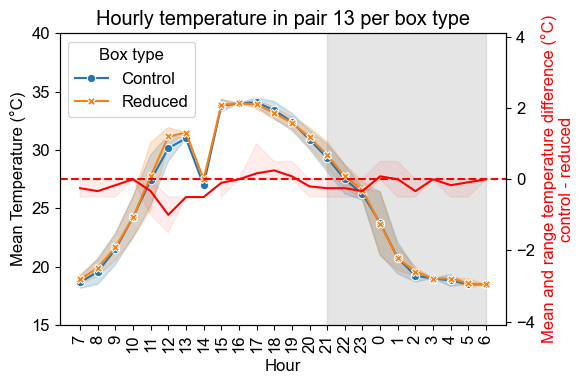

Supplement: Supplementary file 1 — Supplementary file1 (DOCX 5650 KB) [file 442_2025_5700_MOESM1_ESM.docx]
